# EXAMINING THE ASSOCIATION AMONG ADLS, DEMOGRAPHICS, WITH FOOD SECURITY ADULTS AT SENIOR CENTERS IN FULTON COUNTY, GA

**DOI:** 10.1093/geroni/igad104.3316

**Published:** 2023-12-21

**Authors:** Kehinde Akintola, Sophia Foley, Angelique Willis, Suhasini Ramisetty-Mikler, Miranda Cook, Kellie Mayfield

**Affiliations:** Georgia State University, Atlanta, Georgia, United States; Georgia State University, Atlanta, Georgia, United States; Georgia State University, Atlanta, Georgia, United States; Georgia State University, Atlanta, Georgia, United States; Open Hand Atlanta, Atlanta, Georgia, United States; Georgia State University, Atlanta, Georgia, United States

## Abstract

Food security amongst community-dwelling older adults attending senior centers is not often examined. It is an integral determinant of health because of the impact on multiple health conditions, which are further impacted by functional impairments and co-morbidities that can come with increasing age. Additionally, meal and nutrition education providers do not typically collect this information, which can be used to better provide services to their clients. This study examined the relationship between food security and demographic variables of older adults attending senior centers in Georgia served by nutrition provider Open Hand Atlanta (OHA). One hundred sixty-five senior center attendees were recruited from nine senior centers throughout Fulton County. The age of the participants ranged between 52 to 97, with most identifying as female (86.7%) and African American (79%). Food security (outcome of interest) was measured by the USDA 6-item module. Bivariate associations were tested between food security and ADLs and iADLs (instrumental ADLs) measured by a 15-item list, health conditions, shopping habits, transportation, caregiving, and demographics. Results showed that food security differed based on race/ethnicity X2 (n=165) = 8.94 (df=2), p = .011 and type of insurance X2 (n=100) = 5.95, (df=1) p = 0.015). In addition, those who are under public/Govt. insurance experience a higher proportion of food insecurity compared to those under private insurance (p=.015). Results have implications for nutrition providers when offering additional services to senior centers and the older adults they serve.

